# Cardiac Alterations in Patients with Familial Lipodystrophy

**DOI:** 10.36660/abc.20190016

**Published:** 2020-02

**Authors:** Minna Moreira Dias Romano, Paula Ananda Inês Chacon, Fernanda Naira Zambelli Ramalho, Maria Cristina Foss, André Schmidt

**Affiliations:** 1 Universidade de São Paulo Faculdade de Medicina de Ribeirão Preto - Medicina Interna, Ribeirão Preto, SP - Brazil; 2 Universidade de São Paulo Faculdade de Medicina de Ribeirão Preto - Centro de Cardiologia, Ribeirão Preto, SP - Brazil

**Keywords:** Lipodystrophy, Familial Partial/genetics, Cardiomyopathy, Hypertrophic, Magnetic Resonance Imaging/trends, Metabolic Diseases/complications

## Abstract

Familial lipodystrophy is a rare genetic condition in which individuals have, besides metabolic changes and body fat deposits, a type of cardiomyopathy that has not been well studied. Many of the patients develop cardiovascular changes, the most commonly reported in the literature being the expression of a type of hypertrophic cardiomyopathy. This article, presented as a bibliographic review, reviews the clinical and cardiovascular imaging aspects in this scenario of cardiomyopathy in a rare metabolic disease, based on the latest scientific evidence published in the area. Despite the frequent association of congenital lipodystrophy and ventricular hypertrophy described in the literature, the pathophysiological mechanisms of this cardiomyopathy have not yet been definitively elucidated, and new information on cardiac morphological aspects is emerging in the aegis of recent and advanced imaging methods, such as cardiac magnetic resonance.

## Introduction

Lipodystrophy is a rare disease characterized by the loss of adipose tissue, which may be generalized or partial.^1^ Its etiology may be congenital or acquired and there is a deficiency in the leptin hormone production, making the carriers of this pathology hyperphagic. Due to the absence of energy storage sites, an ectopic deposition of triglycerides occurs in the skeletal muscle and liver.^2^

The reduced ability to store triglycerides and their ectopic deposition are determinant for the predisposition and severity of complications, such as insulin resistance, diabetes mellitus, hypertriglyceridemia, hepatic steatosis^3^ and, recently discovered, cardiomyopathy. Presentations such as left ventricular hypertrophy or even dilated cardiomyopathy have been described in patients with lipodystrophy.

Genetic lipodystrophies can be divided and subdivided into various types, each one with its specific mutation, which determine the most diverse clinical presentations and possible associations with the development of heart disease. Despite that, this condition is extremely rare, with a higher prevalence in populations with high levels of consanguinity.

This paper aimed to describe familial lipodystrophy and its association with the development of cardiomyopathies, in the light of the latest scientific evidence.

### Classification of congenital lipodystrophies

#### Congenital Generalized Lipodystrophy (CGL)

One of the most frequent types of genetic lipodystrophy is the generalized congenital type, characterized by an autosomal recessive disorder, occurring most often in cases of parental consanguinity. This form is present in all geographical regions and, because of the consanguinity cause, it probably has the highest prevalence reported in some regions of Brazil, such as the Northeast.^4^ Individuals with this alteration have an almost total lack of adipose tissue, leading to prominent skeletal musculature regarding its phenotypic aspect. During childhood, many individuals develop hepatosplenomegaly and umbilical prominence; and during adolescence, complications such as diabetes arise.

This syndrome can manifest in many different forms, being related to one of four existing subtypes and, consequently, to the affected chromosome. Among these subtypes, the Berardinelli-Seip syndrome (BSCL) is well-known, described through the scientific collaboration of the great Brazilian researcher W. Berardinelli. Today it is known that this syndrome is identified by a mutation in chromosome 11q13, which encodes the protein seipin, present in the endoplasmic reticulum, being responsible for the formation of lipid droplets and their fusion within adipocytes. Its absence causes a lack of both metabolically active adipose tissue and mechanical adipose tissue since birth, which may lead to mild mental retardation and cardiomyopathies, making it the most severe of the subtypes.

#### Mandibuloacral dysplasia (MAD) - associated with lipodystrophy

This is a type of genetic lipodystrophy, in which the individuals have skeletal abnormalities, such as mandibular and clavicular hypoplasia, associated with skin atrophy, delayed teething, cranial suture closure and joint stiffness. As a common feature of lipodystrophies, MAD leads to metabolic complications such as diabetes, insulin resistance, hypertriglyceridemia, and low HDL-cholesterol levels.

#### Familial partial lipodystrophy (FPL)

Familial partial lipodystrophy is, mostly, an autosomal dominant disorder characterized by loss of upper and lower-limb fat as well as trunk^5^ fat. These patients have normal fat distribution during childhood and begin to have progressive and variable loss of subcutaneous fat during puberty, typically from the extremities, and in varying degrees from the abdomen and chest.

**Figure 1:**
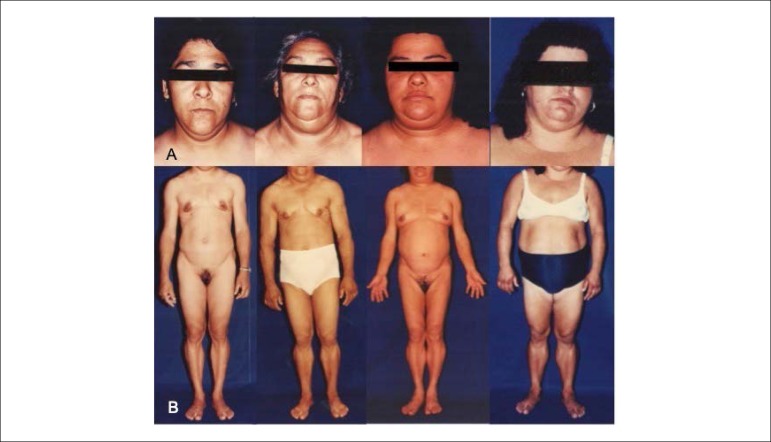
Characteristics of patients with familial partial lipodystrophy. Panel A shows fat accumulation on the face and neck and panel B, fat accumulation in the perineal and intra-abdominal regions.

Many patients, especially females, show fat accumulation in the face, neck and perineal and intra-abdominal regions. (Figure 1) Excess fat accumulation in the dorsocervical (buffalo hump), supraclavicular and submental regions gives these patients a “cushingoid” appearance. In women, there may be masculinization, menstrual irregularity and high prevalence of polycystic ovary syndrome.^5^

Five genes may be involved in the pathophysiology of this type of lipodystrophy, all leading to subcutaneous fat loss in the extremities. The most prevalent form of familial lipodystrophy is autosomal dominant type 2, the first familial partial lipodystrophy more formally described: FPLD2 (Familial Partial Lipodystrophy Type 2), also referred to as variant or Dunnigan Syndrome. This syndrome has a prevalence of 1 in 15 million people, affecting both genders equally. The patients develop several metabolic complications such as dyslipidemia, hypertriglyceridemia and diabetes. In addition, they may manifest varying degrees of myopathy, cardiomyopathy, and other conduction system abnormalities, thus proving to be a multisystem dystrophy.^6^

FPLD2 is characterized by a mutation in the long arm of chromosome 1 (1q21-22) specifically involving lamins A and C or the LMNA gene. Commonly, the mutation that causes FPLD2 affects exon 8 (replacement of arginine by a neutral amino acid at position 482 - R482W), but other mutations in exon 8 and 11 (codon 644 - R644C) have already been described.^6,7^ It has been shown that the LMNA R482W mutation is more associated with muscle and cardiac abnormalities, such as muscular atrophy and dystrophy, cardiac hypertrophy and advanced atherosclerosis.^8^ But the phenotypic differences associated with each specific mutation determinant of FPLD are yet to be elucidated.

Numerous mutations spread throughout the LMNA protein give rise to diseases commonly called laminopathies that affect muscle, heart, fat, cartilage and bone tissues or lead to early aging syndromes.^9^

A-type lamins include lamins A (LMNA) and C (LMNC) that arise from alternative splicing of RNA from the LMNA gene. These proteins are expressed in most cells, and they are located in the nuclear envelope and nucleoplasm and play a relevant role in directing the transcription of heterochromatin located on the periphery of the nucleus.^9^ It is believed that the alteration of these proteins weakens the integrity and structure of the nuclear envelope, which would profoundly deteriorate the structure of the adipocyte nucleus, ultimately leading to premature cell death. In addition, it is known that A-type lamin is capable of interacting with transcription factors such as SREBP1 (Sterol Regulatory Element Binding Protein 1), which is involved in the differentiation of adipocytes.^6,7,9^ Furthermore, it has been observed that type A and C-lamins bind to telomeric sequences, having a role in regulating telomere length.

The main point common to all types of lipodystrophy is the total or almost total absence of adipose tissue, thus compromising the affected individual's storage of triglycerides and their metabolic activity. Before that, most patients clinically manifest muscular appearance, prominent superficial veins, extremity enlargement, acanthosis nigricans, umbilical prominence or hernia, hyperphagia and accelerated growth, menstrual period irregularity , precocious puberty and menarche, among other signs and symptoms that may include cardiac autonomic changes.^10^

One of the key points possibly related to the occurrence of cardiomyopathies in patients with lipodystrophy and also associated with all the described types is the accumulation of fat in ectopic tissues, such as the liver and skeletal muscle. Also, the lower capacity to oxidize and store fat promotes dyslipidemia, insulin resistance, development of diabetes mellitus and its complications, which may involve the cardiovascular system.^10^

### Cardiac impairment in patients with congenital lipodystrophy

The World Health Organization, together with the International Society and Federation of Cardiology, has defined cardiomyopathy as a myocardial disease associated or not with cardiac dysfunction and can be classified according to morphological and physiological changes, such as dilated cardiomyopathy, hypertrophic cardiomyopathy, restrictive cardiomyopathy, arrhythmogenic left ventricular cardiomyopathy, metabolic cardiomyopathy, among others.^11^

Special attention should be paid, in this context, to metabolic cardiomyopathy, which develops before various pathological conditions associated with systemic metabolic disorders, being characterized by structural and functional changes without concomitant coronary artery disease or hypertension.^12^ The condition, known as diabetic cardiomyopathy, is an under classification of metabolic cardiomyopathy and is defined by the presence of myocardial involvement in patients with diabetes, after excluding other causes such as ischemic myocardial disease. In diabetic cardiomyopathy, there may be left ventricular remodeling and dilation, associated with diastolic and, in some cases, systolic dysfunction.^13^

The glucose metabolism impairment present in diabetes is associated with the higher consumption of fatty acids as an energy source, which is justified by the lack of insulin or resistance to it.^13^ The almost exclusive use of this compound leads to excess lipids, which may be accumulated in the heart muscle or diverted to non-oxidative pathways, disrupting normal cell function and causing organ dysfunction and apoptosis, a fact called lipotoxicity.^14^ In addition, permanently hyperglycemia can cause damage to the myocardium through proteins modified by advanced glycation end products, as well as oxygen free radicals, leading to their accumulation and myocardial fibrosis, which can result in dysfunction, initially only diastolic.^13^

Several cardiac alterations have been described in the literature in patients with lipodystrophy, whether solely morphological alterations or alterations associated with cardiac dysfunction, but the pathophysiological basis involved has not been completely elucidated.^10^ Early atherosclerosis, especially in patients with familial partial lipodystrophy, may have a prevalence rate of over 60% and manifests before age 45. With such aggressiveness, the pathophysiological mechanisms involved seem not only to be dependent on metabolic changes, but perhaps on a direct effect of gene mutation on endothelial function.^15^

In a collection of case series of lipodystrophy prior to the year 2000, several cases with hypertrophic cardiac changes have been described, with or without systolic obstruction to ventricular ejection, many with cardiomegaly and associated systemic arterial hypertension (Table 1).^16^

**Table 1:** Case reports of patients with congenital generalized lipodystrophy

Authors/year	Findings
Seip (1959)	3 patients; one with a systolic murmur. 2 had increased blood pressure and cardiomegaly.
Seip (1963)	5 patients, all with cardiomegaly.
Choremis (1965)	1 patient: cardiomegaly and arterial hypertension
Gold et al. (1967)	2 patients (siblings) with moderate cardiomegaly.
Brunzell et al. (1968)	Presence of cystic angiomatosis in patients with CGL.
Montenovesi et al. (1971)	1 patient with cardiomegaly.
Bjorntad et al. (1985)	7 patients, 6 of them with CGL. They had systolic murmur, 3 with left ventricular hypertrophy and 2 with biventricular overload.
Rheuban et al. (1986)	4 patients, all with systolic ejection murmur and non-obstructive hypertrophic cardiomyopathy.
Klair et al. (1993)	1 patient with progressive hypertrophic cardiomyopathy.
Chandalia et al. (1995)	1 patient with 20% obstruction of coronary arteries and presence of atheromatous plaques.
Westvik et al. (1996)	8 patients, 7 of them with CGL. Presence of cardiomegaly and one death secondary to congestive heart failure.
Bjornstad et al. (1996)	8 patients, 7 of them with CGL. Presence of cardiac hypertrophy.
Viegas et al. (2000)	1 patient with hypertension and severe symmetric hypertrophy.

CGL: Congenital Generalized Lipodystrophy. Adapted from Rego AR, et al.^16^

Source: [Adapted from Rego AR, MAG; Faria, CA; Baracho, MFP; Egito, EST; Mesquita, ET; Brandão Neto, J. Alterações cardiovasculares e metabólicas da lipodistrofia generalizada congênita (síndrome de seip-berardinelli)]

After the 2000s, publications on isolated or grouped cases showing an association between lipodystrophy and ventricular hypertrophy^17^ can also been found in the literature, even in very young or still infant patients,^18-20^ and, in some cases, the progression of ventricular hypertrophy has been documented.^21^ In some of these cases identified at early ages, the evolution of cardiomyopathy to global systolic dysfunction in childhood or youth has been documented.^19,21,22^

Hubert Pan et al.^23^ considered that LMNA mutations are generically expressed with muscular dystrophy, lipodystrophies, bone dysplasias, and cardiovascular disease, and, before that, they reported the case of a family with Chinese ancestors with three generations of heart disease, in which 100% of the relatives older than 40 years had the clinical manifestations. The cardiovascular disease shown by the carriers of this mutation consisted of arrhythmias, atrioventricular blocks and dilated cardiomyopathies. Part of these individuals died because of cardiovascular disease.^23^

In 2010, Rêgo et al.^24^ reported a group of 22 patients with CGL2, of which 86.4% had a family history of the disease. Most patients had the common metabolic signs associated with the pathology, such as diabetes mellitus, insulin resistance, acanthosis nigricans, hepatosplenomegaly, elevated fasting blood glucose and triglyceride levels and low HDL-cholesterol levels, which contributed to many patients being diagnosed with metabolic syndrome. On cardiovascular examination, part of the patients had arterial hypertension, 50% of the patients had left ventricular concentric hypertrophy and 4.5% had left ventricular eccentric hypertrophy, but all cases had normal patterns in both left ventricular systolic and diastolic function, when evaluated by conventional echocardiography.^24^

Lupsa et al.^1^ studied 44 patients with lipodystrophy, of whom 31 had CGL. The individuals were submitted to genotypic and phenotypic diagnoses, as well as cardiac morphological and geometric analysis. Of the 31 individuals with CGL, 18 had some degree of ventricular hypertrophy, although none of them had LV systolic dysfunction.^1^ (Table 2)

**Table 2:** Genotypic and phenotypic analysis of patients with CGL

Genetic alteration	Geometrical alteration of the left ventricle	Electrocardiographic and/or functional analysis
CGL1 (AGPAT2 mutation)	9 patients with normal LV mass.	17 ECGs were assessed, 9 of them with abnormalities.
	3 patients with mild hypertrophy.	
	4 patients with moderate hypertrophy.	
	3 patients with severe hypertrophy.	
CGL2 (seipin mutation)	2 patients with normal LV mass.	7 ECGs were assessed, 5 of them with abnormalities.
	2 patients with mild hypertrophy.	
	2 patients with moderate hypertrophy.	
	4 patients with severe hypertrophy.	
LMNA (R133L) mutation	Normal left ventricular mass.	Ejection fraction of 35% and low exercise tolerance.
Unknown mutation	Normal left ventricular mass with concentric remodeling.	

ECG: Electrocardiogram; LV: left ventricle. Adapted from Lupsa BC, et al.^1^

Source: [Adapted from Lupsa BC, Sachdev V, Lungu AO, Rosing DR, Gorden P. Cardiomyopathy in congenital and acquired generalized lipodystrophy: A clinical assessment.]

The pathological analysis of the heart was performed in part of this series; in one of the individuals submitted to heart transplantation because of refractory heart failure, and in two others who died from respiratory failure secondary to pneumonia. Two of the patients had CGL1. In one of them, biventricular dilation and myocyte hypertrophy was evidenced, with the presence of vacuolated subendocardial myocytes, as well as subendocardial and epicardial fibrosis with fat and infiltrated with dispersed lymphocytes. The other individual with CGL1 showed mild left ventricular hypertrophy, especially posterolateral. Finally, in the last individual, who had CGL2, left ventricular hypertrophy and an irregular perivascular and interstitial fibrosis were observed.^1^

**Figure 2:**
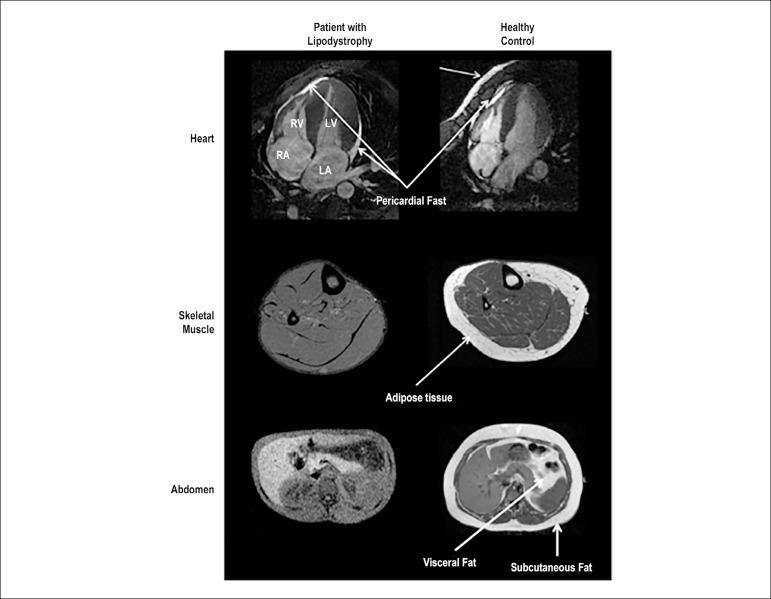
High resolution magnetic resonance images. (Upper) Four-chamber cardiac magnetic resonance images showing pericardial fat in the patient and in the control. (Middle) The control has fat in the chest wall, while the patient does not, demonstrating a general lack of adipose tissue in the patient. (Lower) The liver appears bright because of hepatic steatosis in the patient with lipodystrophy. The general lack of subcutaneous and visceral adipose tissue in the patient with lipodystrophy can be observed. [Adapted from Nelson et. al. Cardiac Steatosis and Left Ventricular Hypertrophy in Patients With Generalized Lipodystrophy as Determined by Magnetic Resonance Spectroscopy and Imaging].^2^

Nelson et al.^2^ studied 5 patients with congenital generalized lipodystrophy (2 of them with CGL1 and 3 with CGL2) and 5 control subjects with similar characteristics of age and body mass. Parameters such as total cholesterol levels and blood pressure were similar in both groups. Individuals with lipodystrophy, however, had low HDL cholesterol levels, high fasting blood glucose levels, three-fold higher circulating triglyceride content, and detection of pericardial adipose tissue by cardiovascular imaging methods (Figure 2). A morphological and functional analysis of the heart of these patients by cardiac magnetic resonance imaging showed an increase in left ventricular mass with a concentric pattern. However, there was no difference between the final systolic and diastolic volume values ​​or left ventricular ejection fraction when compared to a control group.^2^ Thus, in the first clinical study that controlled variables such as blood pressure levels and age between subjects with lipodystrophy and controls, there was still a LV geometric difference between the groups, although both showed no functional alterations.

Sims-Williams et al.^25^ reported the case of a 62-year-old patient with dilated cardiomyopathy and symptomatic heart failure whose twin brother had died from heart failure, as well as their father. The grandfather, on the other hand, had suffered an embolic stroke and also had ventricular hypertrophy. The entire family had an LMNA gene mutation. The echocardiographic evaluation of this individual revealed marked dilation and deterioration of the left ventricular systolic function, with an LV ejection fraction of 18%, associated with a thickness increase in the left ventricular posterior wall. There was also reduction in the right ventricular function and bi-atrial dilation. Based on these facts, the authors hypothesized that most of the cardiomyopathies found in this group of patients also have a familial etiology, and part of them, caused directly by LMNA mutations.^25^

**Figure 3:**
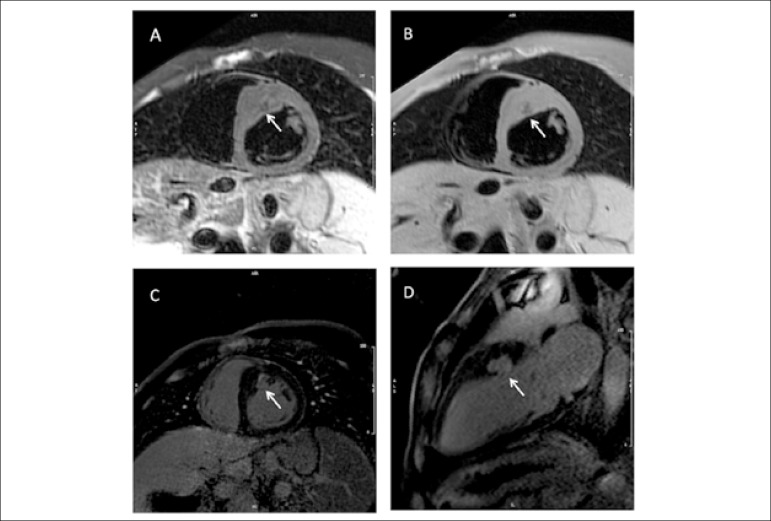
A and B are pre-contrast images. C and D are late post-enhancement images. (A) and (B), both showing a hypodense area (arrow) in the anterior hypertrophic region/anteroseptal region excluding, respectively, the presence of fatty infiltration or edema. (C) and (D) showing late gadolinium enhancement area (arrow) involving the anterior/anteroseptal hypertrophic region and compatible with myocardial fibrosis / necrosis [Adapted from Scatteia et al. Asymmetric hypertrophic cardiomyopathy in generalized lipodystrophy].^26^

Scatteia et al.^26^ described the case of a 30-year-old man with generalized lipodystrophy, diagnosed from birth and with ejection systolic murmur from childhood. Echocardiographic examinations showed asymmetric hypertrophic cardiomyopathy, predominantly in the interventricular septum, mitral regurgitation, but without obstruction of the left ventricular outflow. At the age of 30, the cardiac geometric changes became more evident and ventricular hypertrophy progressed, with an ejection fraction of 71%. With the support of magnetic resonance imaging, it was possible to exclude the presence of myocardial edema, as well as fatty infiltration. This examination revealed a focal area of gadolinium ​​late enhancement, suggesting local myocardial fibrosis (Figure 3). The presence of signs of fibrosis scattered in hypertrophic muscle usually suggests that hypertrophy is primary and not secondary to hemodynamic situations, such as systemic arterial hypertension.^26^

Therefore, as most data in the literature involving lipodystrophy and cardiac geometric and functional changes are based on case reports and case series, the mechanisms involved in this association have yet to be elucidated. The hypothesis of myocardial lipotoxicity is supported by the finding of high triglyceride levels in the hypertrophied cardiomyocytes of some patients, besides the presence of myocardial fat. A probable pathophysiological explanation for lipotoxicity would be a repetitive mechano-sensitive stimulation of elements present in adipogenesis, similarly to patients with preserved residual adipose tissue. In addition, it is also believed that insulin resistance, present in practically all of these individuals, causes an imbalance in the use of substrates by the myocardium, leading to a higher absorption of fatty acids, which may lead to the observed alterations.^2^

Published in 2017, the study by Joubert et. al.,^27^ in an experimental animal model (rodent) of lipodystrophy, was designed to elucidate the pathophysiological basis of myocardial aggression in this disease. Genetically modified mice with no gene for seipin, when compared to controls, showed left ventricular hypertrophy associated with both diastolic and systolic ventricular dysfunction. However, in contrast to what was suggested in other studies, they did not have cardiac triglyceride deposits, contradicting the hypothesis of myocardial lipotoxicity. However, myocardial changes induced in this model correlated with altered glucose metabolism. Based on the findings of this study, it cannot be confirmed that cardiac lipotoxicity is the pathophysiological mechanism involved with hypertrophy and ventricular dysfunction in this disease. But it is quite plausible that the metabolic alteration of glycemic control is indirectly related to transcription factors responsible for the regulation of pro-hypertrophic gene activation.^27^

## Conclusion

Familial lipodystrophy is a rare condition where individuals show, besides metabolic and skeletal muscle changes, adipose tissue alterations, a type of cardiomyopathy. Cardiac changes commonly described in the literature, in case series, show a hypertrophic cardiomyopathy phenotype. The evolution to left ventricular systolic dysfunction can happen in a percentage of cases. There is not enough information to conclude on the frequency of cardiac functional impairment, such as the diastolic dysfunction type, or even incipient systolic changes.

Still, despite the frequent association of congenital lipodystrophy and ventricular hypertrophy, the pathophysiological mechanisms remain unknown. Hypotheses that the altered glucose metabolism caused by the disease is responsible for activation of pro-hypertrophy genes could explain such association.
